# Artificial intelligence technology for myopia challenges: A review

**DOI:** 10.3389/fcell.2023.1124005

**Published:** 2023-01-17

**Authors:** Juzhao Zhang, Haidong Zou

**Affiliations:** ^1^ Department of Ophthalmology, Shanghai General Hospital, Shanghai Jiao Tong University School of Medicine, Shanghai, China; ^2^ Shanghai Eye Diseases Prevention and Treatment Center, Shanghai Eye Hospital, Shanghai, China; ^3^ National Clinical Research Center for Eye Diseases, Shanghai, China; ^4^ Shanghai Engineering Center for Precise Diagnosis and Treatment of Eye Diseases, Shanghai, China

**Keywords:** artificial intelligence, machine learning, deep learning, risk prediction, myopia, classification, semantic segmentation

## Abstract

Myopia is a significant global health concern and affects human visual function, resulting in blurred vision at a distance. There are still many unsolved challenges in this field that require the help of new technologies. Currently, artificial intelligence (AI) technology is dominating medical image and data analysis and has been introduced to address challenges in the clinical practice of many ocular diseases. AI research in myopia is still in its early stages. Understanding the strengths and limitations of each AI method in specific tasks of myopia could be of great value and might help us to choose appropriate approaches for different tasks. This article reviews and elaborates on the technical details of AI methods applied for myopia risk prediction, screening and diagnosis, pathogenesis, and treatment.

## Introduction

Artificial intelligence (AI), first proposed by John McCarthy in 1956, refers to the science and engineering of making intelligent computer programs and is considered one of the key technologies of the fourth industrial revolution. Due to its great potential for automated analysis of medical information and imaging, AI is rapidly developing in the medical field (Gupta et al., 2020). This pattern of automated screening, diagnosis, or risk assessment based on clinical and imaging data has proven to be applicable to a wide range of clinical diseases such as cardiovascular diseases (Wang et al., 2017), neurological diseases (Sarraf and Tofighi, 2016), respiratory diseases (Zech et al., 2018), and malignancies (Coudray et al., 2018), and has a tendency to be translated into real clinical practice. For ocular diseases such as diabetic retinopathy (DR) (Gulshan et al., 2016), age-related macular degeneration (AMD) (Peng et al., 2019), and cataracts (Gutierrez et al., 2022), AI has been used for screening, diagnosis, and other aspects. However, there are relatively few applications of AI in myopia.

Myopia is one of the most common refractive errors. In myopic eyes, the high corneal curvature and long eye axis cause distant objects to be imaged in front of the retina, resulting in blurred vision at a distance and affecting human visual function (Morgan et al., 2012). The current global prevalence of myopia is estimated to be about 28.3%, and this number will grow to 49.8% by 2050 (Holden et al., 2016). The situation is even worse in East Asia (Edwards and Lam, 2004; Han et al., 2019; Ueda et al., 2019; Dong et al., 2020). Online remote learning and working styles have led to a further increase in myopia rates, especially among school-age adolescents (Liang et al., 2021). If left uncontrolled, myopia can progress to high myopia. This will increase the likelihood of developing irreversible fundus lesions or pathologic myopia and is one of the main causes of low vision or even vision loss. In China, myopia prevention and control has become a national strategy, but there are still many challenges to overcome to achieve this goal, which needs the help of novel technology.

Currently, the main challenges faced in myopia are: A) Unclear pathogenesis. It is difficult to objectively quantify the role of many impact factors on myopia, such as genetics, environment, and lifestyle. The morphological changes in eyes are also uncertain; B) Large screening workload. Myopia can only be prevented but not cured, and currently the most effective way is mass screening and follow-up. However, an insufficient number of relevant equipment and ophthalmologists makes it impossible to achieve large population coverage; C) Difficulties in risk prediction. The lack of reliable risk prediction model for high and pathologic myopia, as well as individual differences in the progression of myopia makes it difficult to provide timely intervention; and D) Uncertain efficacy. There is a range of myopia prevention and control methods including outdoor activities, spectacles, corneal contact lenses, atropine, and surgical treatments. Emerging methods include low-intensity red light irradiation. However, it is still a question of how to choose the most appropriate method for each individual.

Various works have been proposed to review the research of AI in myopia (Foo et al., 2021; Du and Ohno-Matsui, 2022; Zhang et al., 2022). To our knowledge, none of the existing work has elaborated technical details of the discussed work, thus fail to make readers more aware of strengths and limitations of each AI method in specific tasks of myopia. This could be of great value to readers with a technical background who are interested in ophthalmic data analysis and myopia. Therefore, our review investigates how AI methods can be applied to address important challenges in the field of myopia and their technical details, with the hope of informing relevant researchers including ophthalmologists and computer scientists.

**FIGURE 1:**
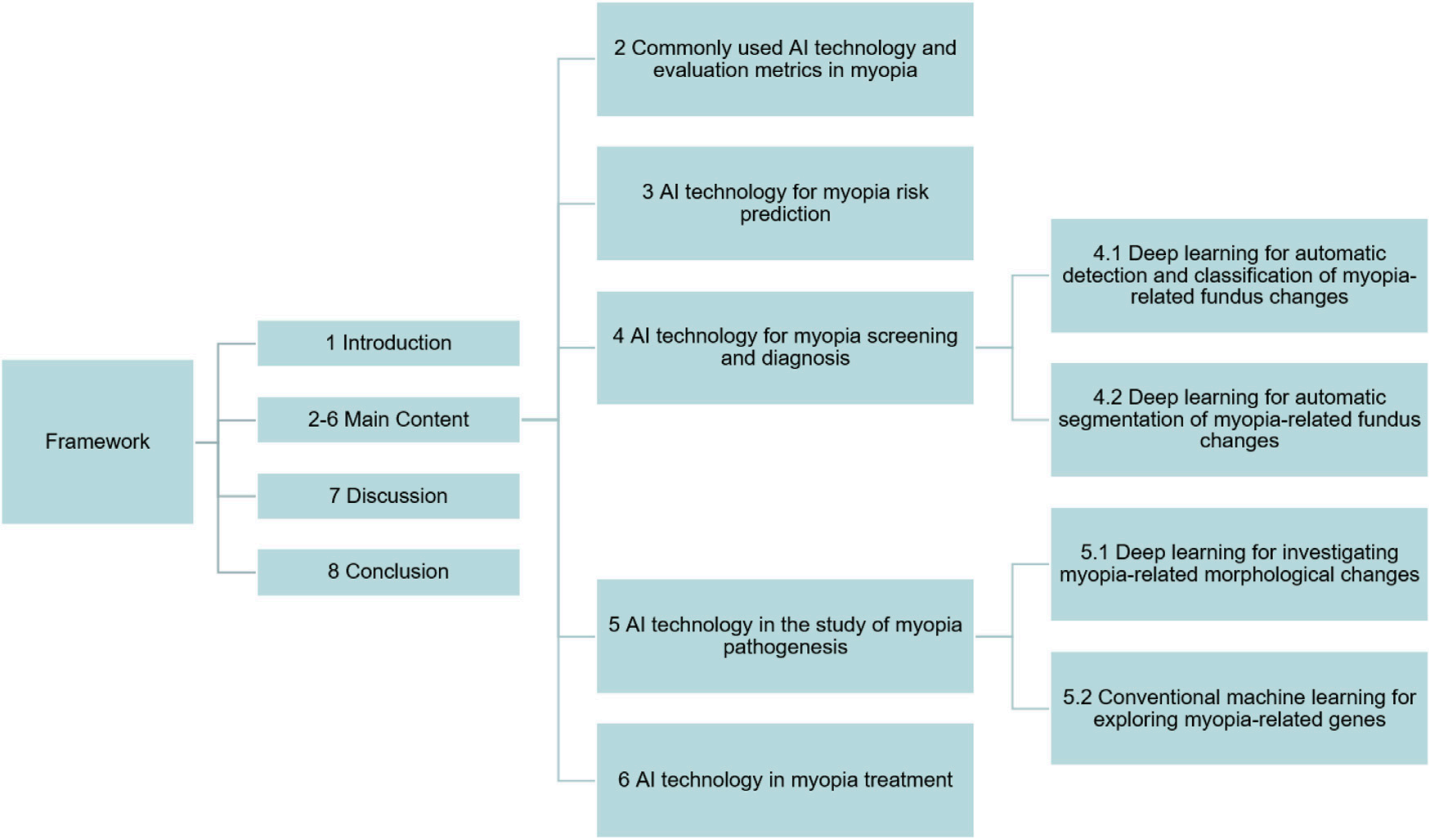
Overview diagram of this review.

The basic framework of this review is depicted in Figure 1. In the second part, we summarize widely used AI technology and evaluation metrics in myopia at present; the following four parts focus on the research progress and technical details of different AI technology in myopia risk prediction, myopia screening and diagnosis, myopia pathogenesis, and myopia treatment, respectively; the seventh part provides a comprehensive discussion of the challenges and future prospects of AI in myopia.

## Commonly used AI technology and evaluation metrics in myopia

In the absence of a universal evaluation benchmark, existing research in myopia does not start with a single AI method, but usually tries several models at the same time and selects the best performing one after parameter tuning and inter-model comparison. Machine learning (ML) is an important branch of AI that refers to methods for training computers to automatically learn relationships between inputs and outputs without explicitly programming them for each situation, and is suitable for analyzing large-scale medical data (Deo, 2015). Conventional machine learning (CML) methods (Chauhan and Singh, 2018) such as linear regression, support vector machine (SVM), and random forest (RF) have been applied in myopia. Newly proposed integrated learning methods such as XGBoost and Gradient Boosting can also been seen (Balyen and Peto, 2019). On the other hand, with breakthroughs in computing power and the introduction of convolutional neural networks (CNNs), deep learning (DL) methods are performing well in the analysis of medical images (Albawi et al., 2017). Some basic deep learning network structures including ResNet, DenseNet, Inception V3, MobileNet, UNet, and VGGNet are widely used in solving problems in the field of myopia. In addition, due to data privacy issues, most myopia studies can only train models based on data from a single center or several centers in the same region, so pre-trained models or transfer learning methods are often utilized to achieve better performance on relatively small datasets.

**FIGURE 2:**
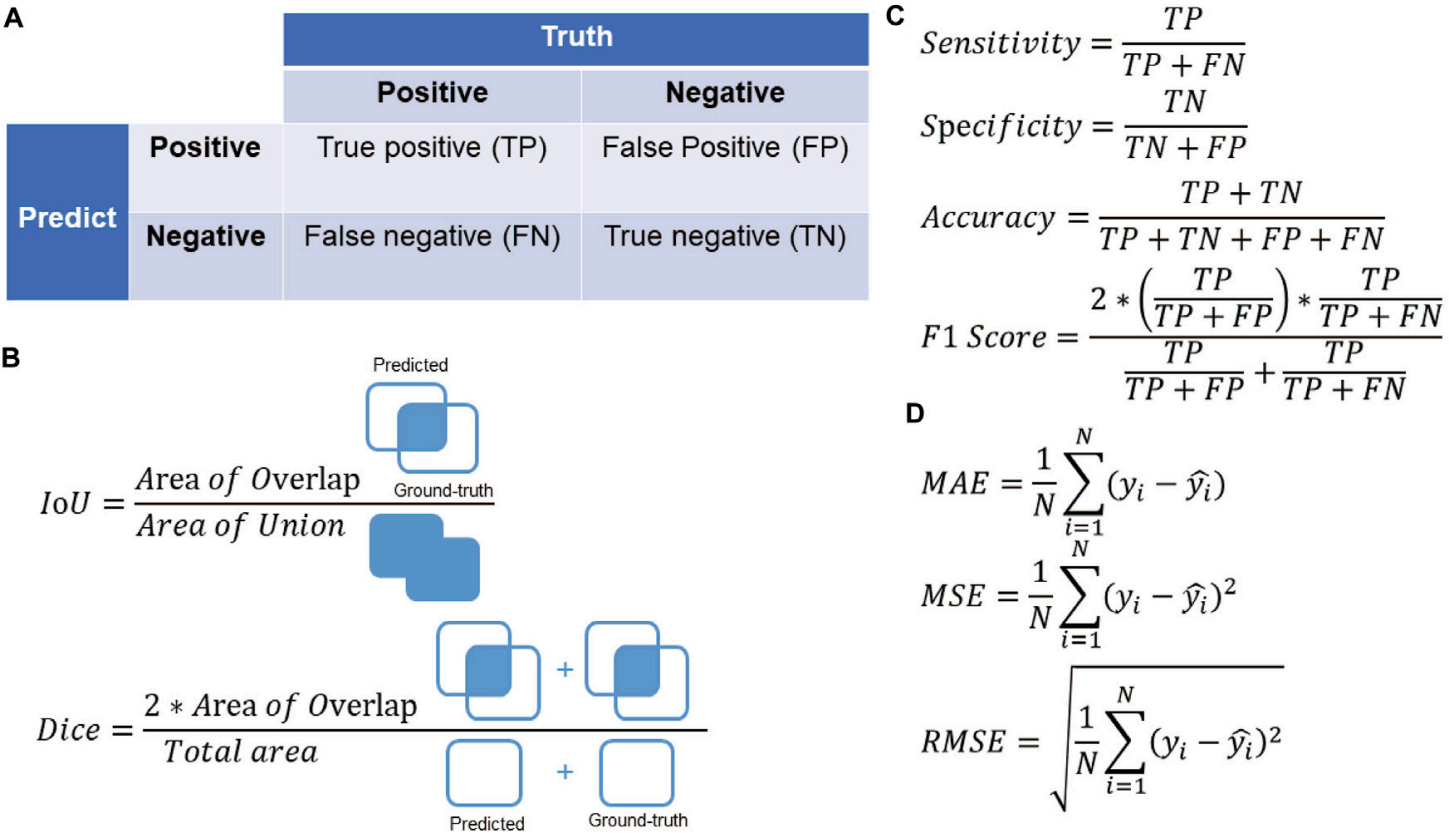
**(A)** The confusion matrix. **(B)** Evaluation metrics for segmentation tasks in the field of myopia. **(C)** Evaluation metrics for classification tasks in the field of myopia. **(D)** Evaluation metrics for regression tasks in the field of myopia. $y_{i}$ is the ground-truth value of sample i and $\hat{y_{i}}$ is the predicted value of sample i .

When evaluating the performance of a model, AI research in myopia often uses the following metrics: For classification tasks such as disease detection and prognosis prediction, metrics calculated from the confusion matrix such as accuracy, sensitivity, specificity, and F1 score are usually used for evaluation. Area under the receiver operating characteristic curve (AUROC) and area under the precision-recall curve (AUPRC) are also commonly used and give a more general idea about the classifier performance, for they do not require a cut-off point; When the task is to derive a prediction region, such as lesion segmentation of fundus pictures, it is often evaluated by Intersection of union (IoU) and Dice similarity coefficient (DSC). These two metrics measure the overlap area between the predicted region and the ground truth; While for regression tasks such as refraction prediction and axial length prediction, the evaluation is often performed using mean absolute error (MAE), mean square error (MSE) and root mean square error (RMSE). The detailed calculation method of these evaluation metrics is presented in Figure 2.

## AI technology for myopia risk prediction

In clinical work of myopia, it is often necessary to evaluate and follow up patients with low to moderate myopia, especially to monitor the visual acuity of the pediatric and adolescent population. This will generate a series of data and records including (Chen et al., 2021): myopia-related risk factors (e.g., near work time, outdoor activity time, genetics, race, gender, etc.), best-corrected visual acuity, refraction, axial length, and some ocular metrics (e.g., intraocular pressure, ocular surface conditions). Analyzing and interpreting these data is a challenge: on the one hand, there is a lack of reliable risk prediction models to determine the progression of myopia patients and their prognosis. On the other hand, given the size of the data and the complexity of a disease like myopia, it is difficult to perform manual analysis.

Conventional machine learning methods have the ability to process large amounts of data in a non-linear way and to extract a large number of potential predictor variables, even though their number may exceed the number of observed variables. This characteristics is suitable for analyzing myopic data (Obermeyer and Emanuel, 2016). Based on the eye and behavioral data from more than three thousand elementary school students, a study by Yang et al. (2020) provided a systematic solution that included feature selection, data cleaning, and model training. A series of protective and risk factors for myopia were screened, and a risk prediction model based on SVM was highly accurate in predicting the occurrence of myopia in the future. Compared to using a single model, Li et al. (2022a) introduced the idea of ensemble learning and constructed a strong classifier by integrating a large number of decision trees as the basic unit. However, there was no significant improvement in the results, which may be related to the dataset and the selection of predictor variables. In addition, the number of samples available for machine learning algorithms has greatly increased in the era of big data, enabling us to train models with sufficient samples. A study that included data from more than 600,000 refractive examinations confirmed the value of large data volume in improving machine learning performance (Lin et al., 2018). However, clinical data collected in real-world settings are often biased, and different studies set up validation sets in different ways. Reasonable evaluation of the performance of different models and improving their generality are issues that need to be solved.

Besides predicting refractive data, the choice of the target variable can also vary according to clinical need. For children wearing orthokeratology lens, changes in corneal curvature make refractive examinations inaccurate in assessing myopia progression, while axial length is more reliable. Tang et al. (2020) showed that robust regression model was able to achieve an accurate prediction of axial length growth. Also, with the use of electronic medical records at all levels of medical institutions and the establishment of standardized information management systems, information and data interoperability will be realized among institutions. With more information that can be mined, not only can the model performance be improved, but also more application scenarios will emerge. Table 1 summarizes the discussed conventional machine learning methods for myopia risk prediction.

**TABLE 1:** Summary of CML methods for myopia risk prediction.

Research	Tasks	AI technology	Accuracy	Sensitivity	Specificity	AUC	MAE	R^2^
Yang et al. (2020)	Prediction of the onset of myopia in primary school students	GBRT, SVM	0.93	0.94	0.94	0.97	—	—
Li et al. (2022)	Prediction of the progression of myopia	RF	0.8–0.9	—	—	—	<0.05D	—
Lin et al. (2018)	Prediction of the onset of myopia in adolescents	RF	—	—	—	0.802-0.888 (8 years in advance)	0.678-0.879 (8 years in advance)	0.743-0.912 (8 years in advance)
Tang et al. (2020)	Prediction of axial length growth	Robust linear regression	—	—	—	—	0.293	0.86

GBRT, gradient boosting regression tree; SVM, support vector machine; RF, random forest.

## AI technology for myopia screening and diagnosis

As myopia progresses further, the axial length (AL) increases, the optic disc begins to tilt and twist, and irreversible retinal chorioretinopathy may develop. These are some of the fundus lesions associated with high myopia. Among these patients, about 3.1% eventually develop different types of myopic maculopathy with a characteristic set of pathological changes (Chang et al., 2013). Pathologic myopia and its complications have become the leading cause of blindness in China (Duan et al., 2021) and there are some urgent needs in this area. First, early identification of fundus changes is important. About 14% of myopic patients are highly myopic and at least one fundus examination is recommended annually to assess the condition of central and peripheral retina (Gifford et al., 2019). However, manual interpretation of these images is laborious and even unfeasible. Second, myopia-related fundus lesions are not obvious in their early stages and are difficult to describe or quantify. Doctors with different experience will give different judgments. This is a matter of concern in those districts with little medical care and is not suitable for the promotion of large-scale and standardized screening at the community level.

### Deep learning for automatic detection and classification of myopia-related fundus changes

The detection of fundus lesions and myopia-related complications in high myopia is an important need, for which deep learning methods such as CNNs already have high accuracy (Shao et al., 2021; Sun et al., 2021). Compared to the manual, deep learning methods take only a few hours to a few days in the training phase of the model and can produce instant results when interpreting images. It is even possible to achieve “offline prediction” based on smartphones (Natarajan et al., 2019). The structure of CNNs consists of four parts: preprocessing, feature extraction, classification and special modules representing various novel ideas. The preprocessing part includes noise reduction, enhancement of FP, OCT or other pictures, unifying resolution, focusing on regions of interest (ROIs), etc. The feature extraction part, also known as backbone network, is the core of CNNs. Convolutional kernels are selected to extract image features by convolutional operation on the original input image. The classification part consists of fully connected layers, which convert the output feature map of the last convolutional layer into a one-dimensional vector. The probability of having a certain myopic fundus lesion is obtained using functions such as Sigmoid or Softmax, and compared with a threshold to output the result. There is no fixed definition of special modules, and it is up to the researcher to choose which modules to use and how to use them. Commonly used modules in ophthalmic image processing include attention mechanisms, residual connectivity, and bottleneck structures. In general, the current CNN models applied to myopia are not novel. The fact that in fundus image analysis, the number of pixels in target structures such as lesions, optic cups optic discs and blood vessels is much less than the background and the curved structure of blood vessels (especially capillaries) is often complicated. These traits in ophthalmology imaging result in a difficult sampling problem. Proposing customized backbone networks or special modules based on the characteristics of myopia-related tasks could be a way to further improve the model performance.

Not only the detection, but also the differentiation of diverse classes of myopia-related fundus lesions is challenging. As the difficulty of the task increases, it is generally necessary to increase the depth of the backbone network to ensure that deep features in fundus pictures can be better extracted. However, traditional convolutional neural networks such as AlexNet and VGG16 may suffer from gradient explosion or disappearance when the depth is increased. New methods represented by ResNet (Tan et al., 2021; Ye et al., 2021; Park et al., 2022), InceptionNet (Choi et al., 2021; Li et al., 2022b), and DenseNet (Sogawa et al., 2020) have effectively addressed this problem. Lu et al. (Lu et al., 2021) used ResNet18 as the backbone network to classify lesions in patients with high myopia based on color fundus images. The results showed that the classification accuracy for each lesion specified in META-PM, a widely accepted classification standard for pathologic myopia, was comparable to that of experts, reaching 97.03%–99.41%. On this basis, it is meaningful to engineer relevant algorithms so that these results can truly contribute to clinical and healthcare screening of myopic patients.

In addition to training deeper networks to solve more complex tasks, the application of AI in ophthalmology is mostly carried out by clinicians, focusing more on clinical application value than on algorithms themselves. That is to say, a flexible model that can reduce parameter tuning efforts and match with specific tasks is needed. Google’s EfficientNet (Tan and Le, 2019), proposed in 2019, is a solution based on which Du et al. (Du et al., 2021) trained four bicategorical models to detect four fundus lesions in highly myopic patients, namely diffuse atrophy, patchy atrophy, macular atrophy, and choroidal neovascularization. With EfficientNet-B0 used as basis, models with different parameters can be easily constructed by adjusting depth, width and resolution simultaneously. At the same time, the included MBConv module introduces an attention mechanism that forces the network to pay more attention to the “critical regions” of the image. The results showed that the detection accuracy of this auxiliary classification system for all lesions except choroidal neovascularization was more than 84%, and the overall detection accuracy for myopic macular degeneration was up to 87.53%, whereas the classification accuracy of ophthalmology specialists on the same task was merely 89%. A study by Li et al. (2022c) showed similar results, further confirming the effectiveness of EfficientNet. However, according to Du et al. (2021) , the detection of choroidal neovascularization was only 37.07%, which might be related to the poor visualization of blood vessels in color fundus images (Jiang et al., 2020; Laíns et al., 2021). OCTA can image blood vessels better, but there are currently no studies using AI methods to analyze OCTA images in myopic eyes. Table 2 summarizes the above-presented methods for automatic detection and classification of myopia-related fundus changes.

**TABLE 2:** Summary of DL methods for classification tasks in myopia.

Research	Tasks	AI technology	Accuracy	Sensitivity	Specificity	AUC
Lu et al. (2021)	Detection of PM and classification of MM	ResNet18; FPN-based Faster R-CNN	0.970–0.994	0.684–0.978	0.970–0.995	0.979–0.995
Du et al. (2021)	Classification of MM	EfficientNet	0.875–0.975	0.370–0.872	0.945–0.983	0.881–0.982
Tan et al. (2021)	Detection of high myopia and MM	ResNet101	—	—	—	0.913–0.978
Li et al. (2022)	Detection of tessellated fundus and PM	Dual-stream DCNNs	—	0.811–0.988	0.959–0.996	0.970–0.998
Sogawa et al. (2020)	Detection of MM	VGG16/19; ResNet50; Inception V3; InceptionResNetV2; Xception; DenseNet121/169/210	0.676–0.965	0.906–1.000	0.942–1.000	0.970–1.000
Li et al. (2022)	Detection of four myopic vision-threatening conditions	InceptionResNetV2	—	—	—	0.961–0.999
Choi et al. (2021)	Detection of high myopia	ResNet50; InceptionV3; VGG-16	—	—	—	0.860–0.900
Ye et al. (2021)	Detection of MM	ResNet101	—	—	—	0.927–0.974
Park et al. (2022)	Detection of PM	ResNet18/50; EfficientNet	0.860–0.950	0.850–0.930	0.880–0.960	0.950–0.980

PM, pathologic myopia; MM, myopic maculopathy.

### Deep learning for automatic segmentation of myopia-related fundus changes

Besides the above-mentioned research with direct outcomes, completing semantic segmentation tasks on fundus images of myopic eyes helps us better comprehend the morphological changes (Read et al., 2019). It can also aid in the training of physicians to interpret images (Fang et al., 2022). In labeling the choroid and the layers of the retina in OCT images, Cahyo et al. (2020) took advantage of the multi-scale feature fusion characteristic of UNet, thus preserving more information. UNet is one of the most commonly used models for semantic segmentation of medical images (Ronneberger et al., 2015; Li et al., 2021). It proposes a novel structure called “Decoder-Encoder”: the decoder is used for feature extraction, and the encoder is used for up-sampling and feature fusion, which is very suitable for medical images with simple semantics and fixed structure. The results showed that by fusing shallow features with little semantic information but accurate target location and deep features with rich semantic information but coarse target location, the IoU could reach above 0.90. Accurate segmentation results were obtained even for the thin choroid of highly myopic patients. By using the upgraded version of UNet, namely UNet++, the segmentation of optic disc, retinal atrophy lesions, and retinal detachment lesions was also satisfactory (Hemelings et al., 2021). However, UNet is a standalone network structure that is difficult to combine with other networks. In view of this, Feature Pyramid Networks (FPNs), a module that can be added after many network structures, was proposed in 2017 (Lin et al., 2017). The core idea consists of two: up-sampling deep features and fusing features from each layer at different depths, and performing prediction independently at different feature layers. Lu et al. (2021) applied FPN to the focal segmentation task of myopic macular lesions and showed that the performance could be substantially improved without changing the structure of the original model and with essentially no increase in computational load.

Completing classification or other tasks on the basis of semantic segmentation is a new direction of current research (Shao et al., 2021). Based on the segmentation results of the choroid and retina, Chen (Chen et al., 2022) quantified the thickness of each layer by adding an additional fully connected layer. The retina is histologically divided into ten layers that are only 400–500 microns thick at their thickest point and may be even thinner in myopic eyes. Therefore, their results can help physician improve the accuracy of interpretation. Notably, while not common currently in AI research in myopia, this type of application is widespread in diabetic retinopathy and can change the “end-to-end” workflow (i.e., prediction directly based on entire images). Table 3 summarizes the above-mentioned studies for automatic segmentation of myopia-related fundus changes.

**TABLE 3:** Summary of DL methods for segmentation tasks in myopia.

Research	Tasks	AI technology	Accuracy	Sensitivity	Specificity	IoU	Dice score	F1 score
Cahyo et al. (2020)	Segmentation of choroid in myopic eyes	U-Net	0.99	—	—	0.92	—	—
Lu et al. (2021)	Segmentation of myopic “Plus” lesions	ResNet50; FPN	0.656–0.789	—	—	—	—	0.688–0.889
Chen et al. (2022)	Segmentation and quantification of the choroid in myopic eyes	Mask R-CNN	—	—	—	—	0.938	—
Li et al. (2021)	Segmentation of choroidal sublayers and vessels	U-Net	0.980–0.987	0.699–0.962	0.990–0.999	—	0.699–0.959	—
Shao et al. (2021)	Segmentation of tessellated fundus and calculating FTD	ResNet18; FCN	0.965	0.725	0.961	—	—	—
Hemelings et al. (2020)	Segmentation of myopia-related fundus changes	U-Net++	—	—	—	—	0.93 (optic disc), 0.80 (retinal atrophy), 0.80 (retinal detachment)	0.98 (optic disc), 0.91 (retinal atrophy), 0.70 (retinal detachment)

FTD, fundus tessellated density.

## AI technology in the study of myopia pathogenesis

### Deep learning for investigating myopia-related morphological changes

Artificial intelligence can provide new ideas for morphological changes in myopic eyes. On tasks that ophthalmologists cannot perform (e.g., predicting refraction based on fundus images), deep learning methods can be done with low mean square error based on FP, UWF FP, or OCT images (Varadarajan et al., 2018; Shi et al., 2021; Yoo et al., 2022). One explanation is that the model automatically learns fundus changes that are not visible in the early stages of myopia and uses them for prediction. Considering this, Shi et al. (2021) introduced the gradient-weighted class activation mapping (Grad-CAM) method to find the region most essential for model prediction. The core idea is to calculate the gradient of the previous layer of the fully connected layer (i.e.: the last convolutional layer) with respect to each pixel in the input image, and to draw a heatmap from it. The pixels that have a higher impact on the model prediction are closer to the red color in the heatmap, and the pixels with less impact are closer to the blue color. The results showed that the area of interest was concentrated around the optic disc as well as the macula, suggesting a potential relationship between early morphological changes in this region and myopia.

In many mammalian models, choroidal thickness (ChT) can rapidly change in both directions when images are focused anteriorly (myopia) or posteriorly (hyperopia) to the retina before axial changes (Read et al., 2019). Studies have confirmed that the choroid undergoes histological changes before the retina in highly myopic eyes (Jonas and Xu, 2014; Zhou et al., 2017). The choroid can also influence choroidal neovascularization and scleral growth through the secretion of growth factors (Nickla and Wallman, 2010; Scherm et al., 2019) which in turn affects the progression of myopia. However, the choroid is the middle layer of the eye wall and cannot be viewed with the naked eye through fundus images. To investigate the choroidal changes, Sun et al. (2021) applied radiomics methods to the optic disc region. Features were automatically extracted from fundus images using PyRadiomics program, followed by LASSO regression to filter the most predictive features and eventually, a novel optic disc imaging metrics was constructed. The results showed that AI methods can effectively predict ChT based on fundus images rather than OCT images, which facilitates the assessment of early pathological changes in highly myopic eyes and guides early diagnosis and treatment.

### Conventional machine learning for exploring myopia-related genes

Through the use of molecular techniques such as linkage analysis, candidate gene analysis, genome-wide association studies (GWAS) and next-generation sequencing (NGS), many new genes and chromosomal loci associated with myopia have now been identified. Representative studies include CREAM (Verhoeven et al., 2013) and 23andME (Tedja et al., 2018). However, these genes can currently explain less than 10% of the genetic variation in myopia (Han et al., 2022). Considering the size and high dimensional characteristics of this data type, conventional machine learning methods are suitable to transform it into valuable knowledge. Ghorbani Mojarrad et al., 2018 used data from CREAM and 23andME to screen for differential genes and calculate a genetic risk score (GRS), which was used as a variable to construct linear models. The results showed that the inclusion of the GRS significantly improved the performance of models in determining the occurrence of myopia compared with using the number of myopic relatives (NMP) alone (*p* < 0.0001). The model incorporating GRS better estimated refractive error at 7 (R2 = 3.0% vs. 3.7%) and 15 (R2 = 2.6% vs. 7.0%) years old compared to using only age, sex, and NMP, but the improvement was still unsatisfactory, as supported by the results of Chen et al. (2019) . One possible reason is that the current understanding of myopia-related single nucleotide polymorphisms (SNPs) and gene-environment interactions is still limited. As for deep learning methods, deep neural networks (DNNs) and recurrent neural networks (RNNs) can be used for tasks such as variant calling, genome annotation, mutation classification, and “phenotype-genotype” correspondence (Dias and Torkamani, 2019) but have not yet been applied in myopia.

## AI technology in myopia treatment

As mentioned above, the mechanisms of myopia onset and progression are still unclear, thus the methods of myopia prevention and treatment are constantly being updated. For non-progressive myopia (i.e., people with slow progression of myopia and progression≤0.50D/year), available correction methods include spectacles, corneal contact lenses, and surgery (e.g., laser keratomileusis, implantable collamer lens (ICL), posterior scleral reinforcement). For progressive myopia (i.e., those with rapid myopic progression and progression≥0.75 D/year), available control measures include orthokeratology lens, spectacles with multi-point myopic defocus technique or point diffusion technique, medications (e.g., atropine, pirenzepine, 7-methylxanthine), low-energy red light irradiation, and a combination of above methods.

When choosing orthokeratology lens, less trials can help reduce the chance of ocular infections (Kam et al., 2017). The use of conventional machine learning methods can provide an accurate estimate of the proper alignment curve (AC) curvature of the lens. The results of Fan et al. (2022) showed that models such as SVM and Gaussian process had a better fitness with R-squared (R^2^) up to 0.73–0.91. By using different kernel functions, SVM can also assist in the prediction of two important parameters of orthokeratology lens: return zone depth (RZD) and landing zone angle (LZA). The R^2^ can reach above 0.80 and 0.90, respectively (Fan et al., 2022). AI technology also have applications in refractive surgery. Methods like random forest, gradient boosting, XGBoost, and SVM regression (SVR) can assist in implant size selection and arch height prediction in ICL surgery (Kamiya et al., 2021; Kang et al., 2021; Shen et al., 2021). Proper sizing ensures a safe postoperative ICL dome and reduces complications such as angle-closure glaucoma and anterior subcapsular cataract. Using an artificial neural network (ANN) containing dual hidden layers and boosting strategy, Cui et al. (2020) developed an assistance system for the design of SMILE surgical parameters. The postoperative corrected distance visual acuity (CDVA) is similar to the preoperative CDVA, but the postoperative uncorrected distance visual acuity (UDVA) is greater than the preoperative one. This result demonstrated that while AI did not significantly differ from experts in terms of safety, they did increase in terms of effectiveness. However, these studies have made simplifications to clinical need, such as considering only two ICL sizes and converting the regression problem into a classification problem. This may improve model performance but also results in some limitations.

In addition, Wu et al. (2020) retrospectively analyzed a cohort of patients with topically applied atropine for myopia control. They used multiple conventional machine learning methods to predict IOP at the endpoint based on 19 variables, and the best performing XGBoost algorithm had an RMSE of up to 2.2604 mmHg, showing potential in predicting efficacy as well as potential side effects of atropine. Fewer studies have used AI in this area, possibly because cohort data for myopia are more difficult to collect compared to cross-sectional studies. Table 4 summarizes the aforementioned AI-related studies for myopia treatment.

**TABLE 4:** Summary of AI technology for myopia treatment.

Research	Tasks	AI technology	Accuracy	AUC	MAE	RMSE	R^2^
Fan et al. (2022)	Estimation of the AC curvature in orthokeratology lens fitting	Robust linear regression; SVM; Bagging decision trees; Gaussian process	—	—	0.263–0.507	0.373–0.680	0.73–0.91
Fan et al. (2021)	Prescribing CRT lens parameters in adolescents with myopia	Gaussian process; Robust linear regression; SVM	—	—	0.386-0.979 (for LZA); 5.326-8.644 (for RZD)	0.556-1.214 (for LZA); 6.883-10.998 (for RZD)	0.693-0.866 (for LZA); 0.964-0.975 (for RZD)
Shen et al. (2021)	Prediction of the postoperative ICL vault	RF; Gradient Boosting; XGBoost	0.802-0.828 (vault prediction); 0.815-0.822 (ICL size prediction)	0.718–0.765	—	159.03–162.53	0.285–0.315
Kang et al. (2021)	Prediction of the postoperative ICL vault	XGBoost; Light GBM; RF; SVM	0.759 (internal validation); 0.674 (external validation)	—	106.88 (internal validation); 143.69 (external validation)	140.14 (internal validation); 186.29 (external validation)	—
Kamiya et al. (2021)	Prediction of postoperative ICL vault	SVR; Gradient Boosting; RF	—	—	99.6–131.4	—	—
Cui et al. (2020)	Prediction of SMILE nomogram	ANN	—	—	0.066–0.114	—	0.9645
Wu et al. (2020)	Prediction of IOP in children with myopia treated with topical atropine	MARS; CART; RF; XGBoost	—	—	0.778–0.867	2.260–2.432	—

AC, alignment curve; CRT, corneal refractive therapy; LZA, landing zone angle; RZD, return zone depth; SVR, support vector regressor; SMILE, small incision lenticule extraction; ANN, artificial neural network; MARS, multivariate adaptive regression splines; CART, classification and regression tree.

## Discussion

Artificial intelligence-enabled intelligent ophthalmic devices are an important solution to the lack of ophthalmic medical resources (especially in primary hospitals), but the area of healthcare has its unique concerns. To apply AI methods in the process of real myopia clinical practice, we believe that the following aspects should be focused on.

Firstly, physician and patient acceptance is a challenge. Scheetz et al. (2021) showed a high rate of patient satisfaction with AI technology for ophthalmic screening, but Lin et al. (2022) found that residents were “algorithm aversion” and expected more physician involvement in eye screening services. Explainable artificial intelligence (XAI) is a potential solution to open the “black box” and gain the trust of patients. When detecting myopic macular lesions using OCT images, deep learning models can be trained using soft labels and output the probability of belonging to each lesion category rather than predicting a particular category, which has been shown to yield satisfying results (Du et al., 2022). Other visualization methods, such as the occlusion test (Zeiler and Fergus, 2014), saliency maps (Simonyan et al., 2013) and gradient-weighted class activation maps (Grad-CAMs) (Selvaraju et al., 2017) can also retrospectively analyze the prediction process of neural networks and highlight important regions relevant to decision making, thus improving interpretability. AI studies on other ocular diseases often choose to publish their heatmap results (Brown et al., 2018; Keel et al., 2019) and many current studies in myopia are gradually starting to take this on board.

Secondly, it is important to accurately evaluate the performance of AI methods from a technical point of view. Existing AI studies in myopia do not have directly comparable results due to the difference in datasets and the way training set/test set were selected. In this regard, some studies have thought beyond the perspective of clinical applications to the perspective of computer science and have done some “benchmark work” in diabetic retinopathy (Li et al., 2019): by establishing a multicenter, well-labeled dataset and conducting repetitive tests using several state-of-the-art algorithms on same tasks, a benchmark of algorithm performance could be established and serves as a reference for further development of relevant evaluation systems. This can be borrowed to myopic AI research to help address the critical question of how to evaluate whether the performance of an AI method is good enough.

**FIGURE 3:**
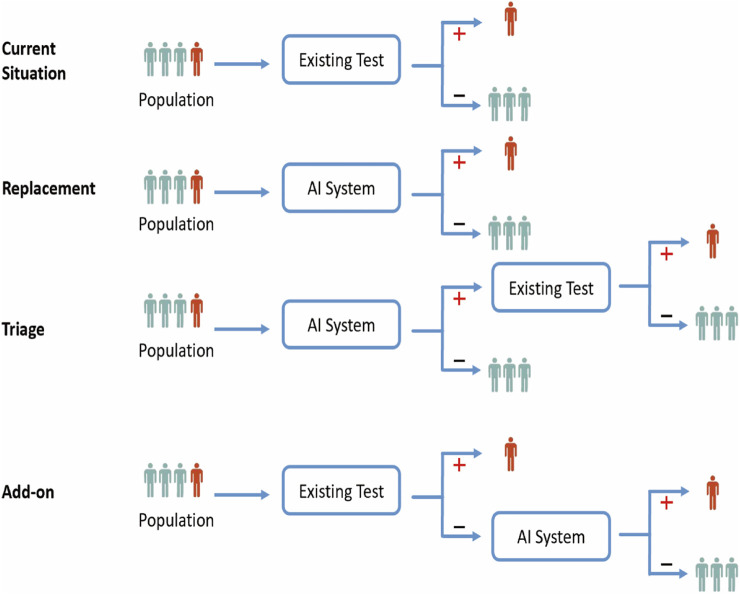
Three ways to integrate AI technology into existing clinical practices.

Lastly, AI should not only continue to improve its performance on evaluation metrics, but also be organically integrated with clinical practice in myopia to achieve a better visual health system. Regarding how to integrate a new technology into existing clinical practice, Bossuyt et al. (2006) summarized three possible ways (Figure 3): replacement, triage, and add-on. For the application of AI in myopia, we believe that the “Triage” and “Add-on” ways are viable and valuable: the former uses AI as the most basic diagnostic classification tool that can serve as a referral for large-scale primary ophthalmology screening or as an “opportunistic screening” in non-ophthalmology clinical work; The latter uses AI in parallel with or after the clinician’s diagnosis to serve as an assistant in tasks like segmenting the layers of the retina or measuring thickness on OCT images for classifying pathologic myopia, as mentioned earlier. As for the “Replacement”, AI algorithms are used to replace clinicians in clinical diagnostic tasks, which is generally only applicable to tasks that are simple enough or where the AI performs absolutely better than the physician. This requires rigorous validation and is not common in the field of myopia.

## Conclusion

The application of AI in the field of myopia is impressive, and its performance holds promise to replace traditional computer-aided diagnostic systems (CADs). The results of this review suggest that AI has been applied to tackle some of the key challenges in myopia clinical practice. However, AI research should not simply be about applying models to various tasks and more attention needs to be paid to those technical problems that have yet to be solved. In the future, more technical approaches need to be proposed according to the characteristics of each task. It is promising that more AI approaches will be deployed as stable and efficient diagnostic systems for practical clinical diagnosis.
